# Effect of Icosa-11,14,17-Trienoic Acid from *Setipinna phasa* Oil on Lipogenesis and Adipose Inflammation on Mice with High Fat Diet Induced Obesity

**DOI:** 10.3390/metabo16060384

**Published:** 2026-06-01

**Authors:** Titli Panchali, Riya Kar, Pipika Das, Ananya Dutta, Manisha Phoujdar, Kuntal Ghosh, Shrabani Pradhan

**Affiliations:** 1 Biodiversity and Environmental Studies Research Centre Affiliated to Vidyasagar University, Midnapore City College, Bhadutala, Paschim Medinipur 721129, West Bengal, Indiadaspipika191@gmail.com (P.D.); ananyadutta457@gmail.com (A.D.);; 2 Central Research Laboratory, Department of Paramedical and Allied Health Sciences, Midnapore City College, Bhadutala, Paschim Medinipur 721129, West Bengal, India; 3 Department of Biological Sciences, Midnapore City College, Bhadutala, Paschim Medinipur 721129, West Bengal, India; micro.kuntal@gmail.com

**Keywords:** adiposity, hypertrophy, cytokines, energy, lipid, fatty acid

## Abstract

**Background/Objectives**: Obesity is a complex disease involving the accumulation of an excessive amount of body fat. It is a condition that develops when energy intake and expenditure are out of balance. Inflammation and hypertrophy are caused by the storage of too much white adipose tissue, resulting in adiposity, which also secretes several pro-inflammatory cytokines. Several marketed drugs used to treat obesity have many side effects from long-term ingestion. Other therapeutic compounds from marine sources have already been established for treating obesity. In this paper, the main aim is to establish the anti-obesity effect of derived omega-3 fatty acids, i.e., 20:3(n-3)11-14-17 Icosa Trienoic Acid from *Setipinna phasa* oil. **Methods**: In the present investigation, inbred male Swiss albino mice were segregated into six categories as Control, Positive Control, Obese Control, and 20:3(n-3)11-14-17 Icosa Trienoic Acid treated groups with three different doses: Treatment 1, Treatment 2 and Treatment 3. To establish the potentiality of extracted fatty acid, different parameters would be considered, such as body weight, lipid composition and different obesity and obesity-associated inflammation markers. **Results**: After the isolated compound from *Setipinna phasa* oil was applied to the treated mice group, it decreased their body weight and serum lipid profile by 39.05%, 62.69%, 62.72%, and 78.46% compared to obese mice. They also had lower levels of uric acid, Serum Glutamic-Oxaloacetic Transaminase, Serum Glutamic Pyruvic Transaminase, and Alkaline Phosphatase, at 67.52%, 57.09%, 64.80%, and 43.99%, than the obese group. Accordingly, the treated group’s expression of genes linked to obesity and pro-inflammatory cytokines was downregulated. The isolated compound affected both anti-inflammatory and anti-obesity markers’ increased expression. **Conclusions**: After the experiments, it was found that the possibility of using fatty acids might be helpful as an anti-inflammatory and anti-obesity therapeutic strategy. This therapeutic strategy will be cheap and cost-effective.

## 1. Introduction

Obesity is a major public health problem day by day due to unhealthy eating habits, especially a combination of excessive fatty and carbohydrate food intake, lack of physical activity, and genetic susceptibility, thereby causing a lack of energy utilization due to the lethargy of the individual [1]. Slow metabolic rate is also responsible for obesity because some obese persons consume little but gain weight heavily [2]. Obesity is defined as the deposition of a large amount of white adipose tissue (WAT) in different regions of our body. Because of the distribution of fat in the body, obesity has different types—either around the waist and trunk (abdominal, central, or android obesity) or peripherally around the body (gynoid obesity)—that have important health implications [3]. It is a medical condition that impairs metabolism, which raises the risk of numerous metabolic disorders, as well as other illnesses and health issues. Obesity raises the risk of developing Type II Diabetes Mellitus (T2DM); cardiovascular diseases (CVD) like hypertension, stroke, and coronary heart disease; gallbladder disease; some cancers (endometrial, breast, prostate, and colon); and non-fatal conditions like gout, respiratory issues, gastroesophageal reflux disease, osteoarthritis, and infertility [4], as well as rheumatoid arthritis, which may cause insulin sensitivity, and early atherosclerosis.

When energy intake exceeds energy expenditure, adipocytes begin to grow. Triacylglycerol, the body’s largest energy store, is processed by adipocytes, which are essential to energy homeostasis in mammals. Mesenchymal stem cells commit to preadipocytes, and preadipocytes differentiate into (adipokine-secreting) adipocytes during the closely controlled process of adipogenesis. Gene expression patterns shift throughout cellular differentiation, and multipotent gene expression modifies these patterns to become cell-type-specific gene expression. Therefore, transcription factors are crucial for adipogenesis. Transcription factors Peroxisome Proliferator-Activated Receptor γ (PPARγ) and Peroxisome Proliferator-Activated Receptor α (PPAR-α) [5] are the main regulators of adipogenesis. Growth arrest, morphological alteration, high expression of lipogenic genes, and the synthesis of adipokines such as Tumor Necrosis Factor- α (TNF-α), leptin, and adiponectin are the main characteristics of differentiated adipocytes. Few studies indicate that adipocyte hyperplasia, which is linked to a higher risk of metabolic problems, may transpire after hypertrophy. As a form of extra energy, triglycerides are stored mostly in the adipose tissue. Adipose tissue also includes a variety of cell types, including immunological, endothelial, and preadipocytes. In a positive energy balance condition, when energy is required between two meals or during physical exercise, triglycerides convert into free fatty acids in adipocytes through lipolysis. Free fatty acids (FFAs) are transported to other tissues to be used as an energy source. FFAs are essential for the emergence of metabolic disorders associated with obesity, especially insulin resistance. These are responsible for cytokine production of macrophages [6] such as Monocyte Chemotactic Protein 1 (MCP-1), TNF-α, IL-1, IL-6, and IL1-β; thus, it interferes with adipose tissue inflammation, which disrupts many obesity-associated metabolic issues. These are proven in few publications [7,8].

Lipids are an essential macronutrient in the human diet since they provide a large proportion of energy. Many of us are not aware of the actual type and proportion of fatty acid that causes disruption of normal metabolism. Studies have indicated that while monounsaturated fatty acid (MUFA) and polyunsaturated fatty acid (PUFA) are beneficial to health, saturated fatty acids (SFAs) are not safe [9]. Diets rich in marine sources, i.e., fish oil composed of omega-3 fatty acids, such as linoleic acid, Eicosapentaenoic Acid (EPA), and Docosahexaenoic Acid (DHA), have potential cardioprotective, anti-diabetic, anti-hypertensive, and hypolipidemic effects, as well as anti-inflammatory and anti-obesity effects [9,10,11]. The majority of people enjoy eating fish, particularly marine fish. As a result, a unique cuisine that includes foods high in unsaturated fatty acids is essential. People have unknowingly adopted a good eating habit that helps them maintain their weight and general well-being by including PUFA in their diet. However, the potentiality of 20:3(n-3) icosa-11,14,17-trienoic acid (11,14,14-ITA) derived from *Setipinna phasa* oil has not been established to date. So, our aim is to establish the anti-obesity effect of derived 20:3(n-3) 11-14-17 Icosa Trienoic Acid from *Setipinna phasa* oil. Thus, in high-fat-induced mice, we investigated the hypothesis that the compound 20:3(n-3) 11,14,14-ITA would reduce obesity and lessen obesity-induced inflammation. Three cis-double bonds are present at positions 11, 14, and 17. It is an omega-3 fatty acid. It is a (11Z,14Z,17Z)-icosatrienoate acid.

## 2. Materials and Methods

### 2.1. Animal Experiments

The animal study was approved by the Institutional Animal Ethical Committee (2256/PO/Re/S/23/CCSEA) MCC/IAEC-SP/10/23-002 of Midnapore City College. Five-week-old Swiss male albino mice weighing 15 ± 2 g were purchased from Saha Enterprise, Kolkata, West Bengal, India. For one week, the mice were acclimated to certain environmental conditions, which included a temperature of 28 ± 2 °C, a 12 h light/dark cycle, and a humidity of 50 ± 5%. The animals were given normal chow food (Hindustan Lever, Mumbai, India) and sterile water ad libitum. Thirty-six mice were separated into six groups, and each group contained six mice; they were treated with different doses of 20:3(n-3) 11-14-17 ITA (Table 1). Mice were given a no-fat diet (NFD) (C; 15% fat, 65% carbohydrate, and 20% protein, methylcellulose (cat# GRM7261-500G)) along with sterile water for sixteen weeks. The OC group had an unlimited supply of water along with high-fat diet (HFD) consisting of 17.8% carbohydrates, 22.2% protein, and 60% fat from lard for sixteen weeks [12]. After twelve weeks of HFD, the treatment was given orally for four weeks, one time per day, after obesity induction. 11-14-17-ITA was purchased from Sigma Aldrich (USA, St Louis, Cat#55682-88-7).

All experiment was carried out in compliance with all applicable standard rules and regulations.

### 2.2. Food Intake Pattern, Body Weight (B.W.) and Body Mass Index (BMI)

The mice in this study had weekly body weight measures taken in addition to their final body weight. Amount of food intake was measured daily. Leftover food was weighed on a twice-daily basis. Food intake pattern was analyzed to observe the link between overgrowth and high-fat diet. A vernier caliper (Central; model # 6420) was used to measure the nose-to-anal distance to within 0.1 mm to compute body length. The conventional method was used to calculate the body mass index, and the findings were represented in g/cm^2^ [12].

### 2.3. Collection of Blood and Tissue Sample

The mice were sacrificed on the last day of the experiment by having their cervical vertebrae dislocated. To separate serum for biochemical estimation, blood was obtained through cardiac puncture and then centrifuged for 20 min at 2500 rpm at 4 °C and stored at −20 °C. The liver, visceral adipose tissue, and subcutaneous adipose tissue were taken, weighed, and stored at −80 °C until analysis. The liver index was calculated by dividing the weight of the liver by the body weight and then multiplying by 100. Each group’s adipose tissues were divided into one part and preserved in 10% formalin before being embedded in paraffin.

### 2.4. Determination of Blood Chemistry

The Coral Clinical System Kit (Bambolin Complex Post Office, Goa, India) was used to determine the serum lipid profile (Cholesterol: cat# 1102040075; Triglyceride: cat# 1102220075) and High Density Lipoprotein (HDL) (Delta Lab, cat# DL1502) in accordance with the manufacturer’s instructions. The Glucose Oxidase-Peroxidase (GODPOD) standard protocol was used to assess the serum glucose level using Autospan Glucose (93DP100-74, Surat, India), and the Coral Clinical System Kit (1101201150, Bambolin Complex Post Office, Goa, India) was used for the Biuret method to estimate the total protein, Serum Glutamic-Oxaloacetic Transaminase (SGOT) (1102200075, Bambolin Complex Post Office, Goa, India), Serum Glutamic Pyruvic Transaminase (SGPT) (1102210075, Goa, India), and Alkaline Phosphatase (ALP) (1102020103, Bambolin Complex Post Office, Goa, India). Serum uric acid was also assessed by the Coral Clinical System Kit (1102260075, Bambolin Complex Post Office, Goa, India).

### 2.5. Histological Analysis

Hematoxylin and Eosin (H&E) and Oil Red O (ORO) staining were performed on dissected, isolated liver, visceral, and subcutaneous WAT samples. These had previously been immersed in paraffin, fixed in a 10% formalin (Merck, Mumbai, India, cat# DB3F730068) solution, and sectioned at a thickness of 5 mm. After that, H&E staining and ORO were carried out. Stained histological sections were subjected to photomicroscopic observation (Olympus, CX21iLED, Gurgaon, Haryana) [13,14]. Using ImageJ software, version 1.53e [15], the diameter and size of lipid droplets of adipocytes in visceral and subcutaneous WAT were determined.

### 2.6. RNA Isolation and Measurement of Obesity Biomarkers by Gene Expression

Visceral adipose tissue was used to isolate total Ribonucleic Acid (RNA), and the HiPura Total RNA Miniprep purification kit (Himedia, India, Cat# MB602-50PR) was used for the specific methodology. RNA isolation method was carried out according to the standard protocol of RNA isolation kit from Himedia. One microgram of total RNA was transformed into cDNA (Complementary DNA) using the Hi-cDNA synthesis kit (Himedia, HiGenoMB, Thane (West), India, Cat# MBT076-100R). Semi qPCR (BioRad, China) was used to assess gene expression. Polymerase Chain Reaction (PCR)PPAR-0 findings were examined on an agarose gel to verify the fragment size and the amplification’s specificity. The relative gene expression levels were normalized by Glyceraldehyde-3-Phosphate Dehydrogenase (GAPDH). The primers that were used in this study were purchased from Bioserve Technologies, Hyderabad, India. The following primer sequences were used in this study (Table 2).

### 2.7. Western Blotting

Isolation of protein from visceral WAT was performed following a previous standard protocol. The Western blot was performed by electrophoresing using Sodium Dodecyl Sulfate–Polyacrylamide Gel Electrophoresis (SDS-PAGE), so a 50 µg protein sample was loaded and then blotted onto a nitrocellulose membrane (BioRad, China). The total procedure of Western blotting was carried out from a previous study (12). Different primary antibodies (mouse monoclonal IgG) associated with obesity and inflammation were used in this study; these are GAPDH (SC-47724), IL10 (Affinity, UK), PPAR-α (SC-398394), Ob (H-5) (SC-393043), PPAR-γ (SC-7273), and Fatty Acid Synthase (FAS) (SC-74540) (Santa Cruz Biotechnology, Santa Cruz, CA, USA) at a 1:1000 ratio. Following three rounds of washing with phosphate-buffered saline with Tween-20 (PBST), the membrane was incubated for one hour at 4 °C with horseradish peroxidase (HRP)-labeled anti-mouse secondary antibody 1:7500 (Abgenex, Bhubaneswar, India). The secondary antibody, goat anti-mouse IgG-HRP (cat#11-301), was purchased from Abgenex (Bhubaneswar, India). We used 3,3′,4,4′-Tetraaminobiphenyl tetrahydrochloride (DAB) to identify specific protein bands. Protein immunoreactivity was measured using ImageJ software.

### 2.8. Statistical Analysis

The data were presented as mean ± SE, n = 6. The variations in different analysis results were scrutinized by two-way ANOVA (Tukey’s test) using Graphpad prism8 statistical software. Significant variation was accepted at the level of 5 and 1% (i.e., *p* < 0.05 and *p* < 0.001).

## 3. Results

### 3.1. Impact of 11,14,17-ITA on Body Weight, Food Intake and BMI

**Figure 1:**
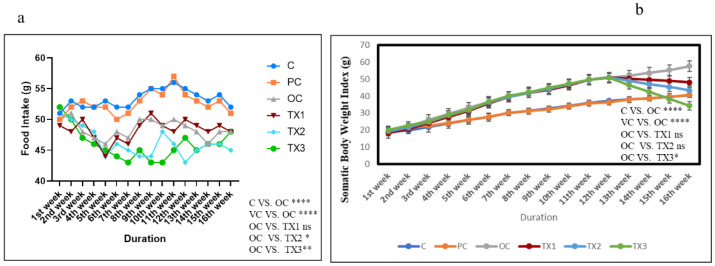
Analysis of food intake (**a**) and somatic body weight changes (**b**) in different experimental groups. Values are expressed as mean ± SEM (n = 6). Two-Way ANOVA followed by Tukey’s multiple comparison test. **** *p* < 0.0001, ** *p* < 0.01, * *p* < 0.05, ns—not significant.

**Figure 2:**
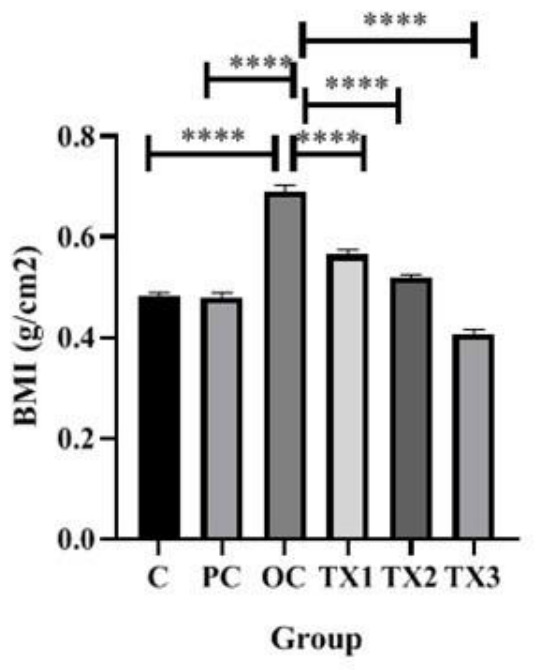
Analysis of BMI in different experimental groups. Values are expressed as mean ± SEM (n = 6). One-Way ANOVA followed by Tukey’s multiple comparison test. **** *p* < 0.0001.

Food intake by obese mice fed HFD was less than that for those fed NFD. However, total energy intake by animals in the NFD group was lower compared to the HFD-fed group. This is because HFD content has more energy due to the presence of lard compared to NFD, which verified that the HFD influenced the appetite of the mice. Nevertheless, there was no discernible difference between the treatment group and the HFD group, suggesting that 11,14,14-ITA had no discernible impact on the mice’s appetite (Figure 1a). Our previous experiments proved how *Setipinna phasa* oil improves obesity by lowering the body weight of obese mice as well as BMI. All the mice groups started off at around the same weights. After one week of consumption of the experimental diet, the body weight starts to increase in the OC and treated groups (TX1, TX2, TX3) compared to the C and PC groups. At the end of the administration, compared with that in the OC group, the final body weight gain and BMI of TX3 had decreased significantly. Between the TX2 and TX3 groups, there was no difference in body weight, but (Figure 1b) in BMI, there was a significant difference between the three treated groups (Figure 2).

### 3.2. Influence of 11,14,17-ITA Administration Against Metabolic Dysfunction-Associated Steatotic Liver Disease (MASLD) and Visceral White Adipose Tissue (vWAT), Subcutaneous White Adipose Tissue (Swat)

**Figure 3:**
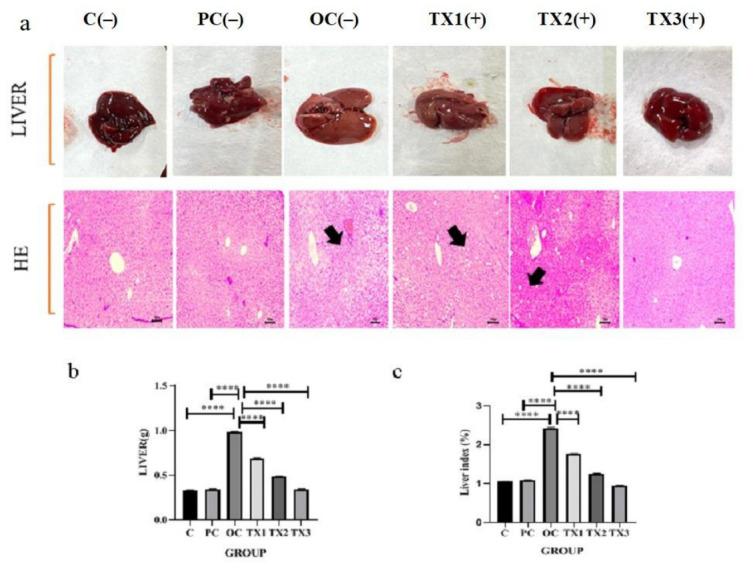
The effect of isolated compound on liver histology by (**a**) HE staining, (**b**) absolute weight of liver, (**c**) liver index. Control (C), Positive Control (PC), High-fat diet-induced obese (OC), High-fat diet-induced obese treated with ITA (TX1, TX2, TX3). The scale bar correspondence to 100 px. Values are expressed as mean ± SEM (n = 6). One-Way ANOVA followed by Tukey’s multiple comparison test. **** *p* < 0.0001.

Steatosis, hepatocellular ballooning, hepatocyte necrosis, and fibrosis are typical histological characteristics of MASLD [16]. From chronic liver disease, MASLD may occur. Previously known as Non-Alcoholic Fatty Liver Disease (NAFLD), this is the new name for the MASLD. The accumulation of fat in the liver, known as MASLD, is frequently connected to metabolic health problems such as insulin resistance, type 2 diabetes, and obesity. Compared to the parameters of NFD-fed mice, the liver sections from HFD-fed mice in the current investigation showed the existence of steatosis, yellowish liver or discoloration of the liver, and ballooning with no signs of cell necrosis and fibrosis. Conversely, HFD-fed mice that received omega-3 fatty acid therapy had no ballooning or steatosis side by side, and the color of the liver in TX2 and TX3 returned to the previous deep red color. Risk of fatty liver and liver dysfunction was decreased gradually at the doses of 1 mg/kg, 2 mg/kg, and 4 mg/kg of B.W. (Figure 3a). Weight of the liver and percentage of liver index were also altered after ingestion of HFD compared to NFD diet, but after treatment with 11,14,17-ITA at different doses, weights were significantly decreased (Figure 3b,c).

**Figure 4:**
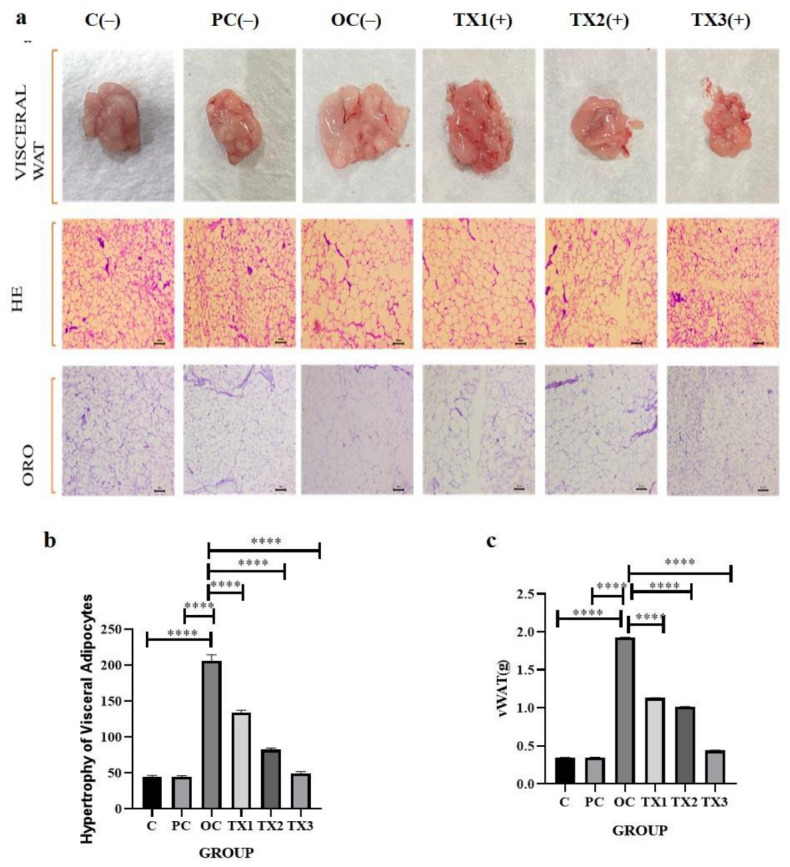
The effect of isolated compound on visceral WAT histology by (**a**) HE and ORO staining, (**b**) vWAT adipocyte size and (**c**) absolute weight of vWAT. Control (C), Positive Control (PC), High-fat diet-induced obese (OC), High-fat diet-induced obese treated with ITA (TX1, TX2, TX3). The scale bar correspondence to 100 px. Values are expressed as mean ± SEM (n = 6). One-Way ANOVA followed by Tukey’s multiple comparison test. **** *p* < 0.0001.

**Figure 5:**
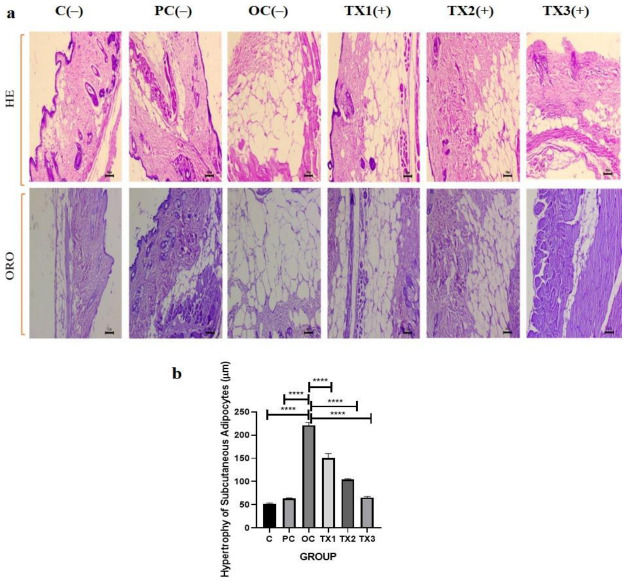
The effect of isolated compound on subcutaneous WAT histology by (**a**) HE and ORO staining and (**b**) sWAT adipocyte size. Control (C), Positive Control (PC), High-fat diet-induced obese (OC), High-fat diet-induced obese treated with ITA (TX1, TX2, TX3). The scale bar correspondence to 100 px. Values are expressed as mean ± SEM (n = 6). One-Way ANOVA followed by Tukey’s multiple comparison test. **** *p* < 0.0001.

The size of fat cells increases with an increase in fat storage. Weights of adipocytes were significantly increased in the HFD group than in those fed with normal diet and the treated group. Pathological investigation using H&E and ORO staining revealed that adipocytes in the HFD group’s vWAT and sWAT were big and possessed fat vacuoles with large inner diameters, although their sizes were not consistent and the cells were not arranged neatly. On the other hand, the NFD group’s adipocytes were grouped tightly and cleanly, and their vacuoles had smaller interior diameters. Compared with the HFD group, the treated group demonstrated a reduction in the vWAT and sWAT’s fat cell size with smaller diameter of fat cells (Figure 4 and Figure 5).

### 3.3. Modulation of Serum Biochemical Changes in Obese Condition with the Help of 11,14,17-ITA

As shown in Table 3, total cholesterol (TC), triglyceride (TG), Very Low Density Lipoprotein (VLDL), and Low Density Lipoprotein (LDL) were significantly increased in the OC group compared to the C and PC groups. Further, 11, 14, 17—ITA gradually reduced in TX1, TX2, and TX3 the serum level of TC, TG, VLDL, and LDL (TX1—25.35%, 19.77%, 19.75%, and 45.17%; TX2—29.79%, 50.83%, 50.84%, and 59.23%; TX3—39.05%, 62.69%, 62.72%, and 78.46%) compared to the OC group. There was also a significant increase in HDL in the groups of TX1, TX2, and TX3 (11.75%, 54.59%, and 69.47%) compared to the OC group. We examined serum fasting blood glucose levels and found significantly higher levels (47.91% and 45.23%) in the OC group than in the C and PC groups. However, mice fed with low and high doses of this omega-3 fatty acid had significantly reduced serum glucose levels compared to OC. The OC group’s serum total proteins decreased significantly as compared to the C and PC groups. After the treatment with various doses of selected fatty acid serum, protein level gradually increased. To find out if there were any hazardous side effects from 11,14,17-ITA treatment, liver function and uric acid tests were conducted. This evaluation is crucial for figuring out whether this fatty acid is safe to use in future applications. Results showed that uric acid, SGOT, SGPT, and ALP did not change significantly between the C and PC groups, but this fatty acid significantly gradually reduced in the treated groups (TX1: 28.53%, 29.9%, 26.83%, 10.24%; TX2: 51.19%, 42.52%, 42.17%, 30.86%; TX3: 67.52%, 57.09%, 64.80%, 43.99%) compared to OC. It was shown that giving the mice under study a low or high dose of this fatty acid for four weeks did not result in any observable harmful or toxic side effects.

### 3.4. Effect of 11,14,17-ITA on Obesity-Related Gene Expression in HFD-Induced Obese Mice

**Figure 6:**
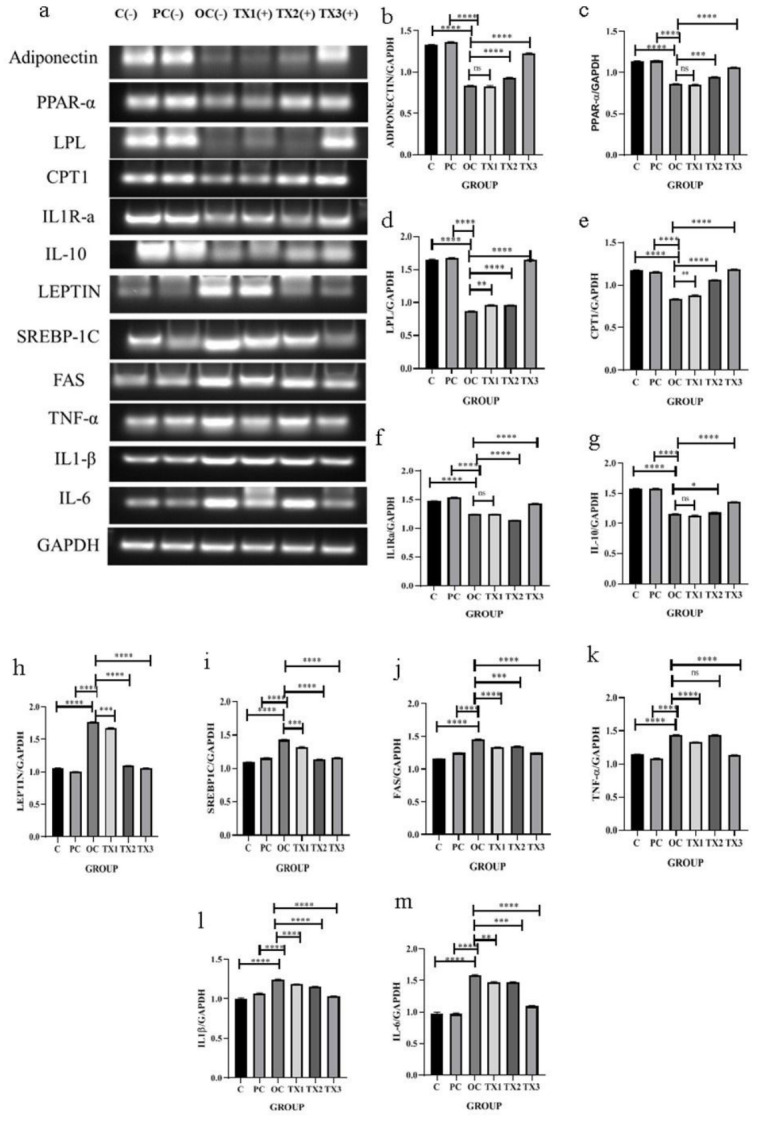
Expression of obesity and inflammatory-related markers by semi qPCR (**a**). Relative gene expression level of adiponectin, PPAR-α, LPL, CPT1, IL1Ra, IL-10 (**b**–**g**) and leptin, SREBP-1C, FAS, TNF- α, IL1β, IL-6 (**h**–**m**) in adipose tissue of male mice in different experimental groups (n = 6) were adjusted to control gene expression level (GAPDH). Values are expressed as mean ±SEM. One-Way ANOVA followed by Tukey’s multiple comparison test. **** *p* < 0.0001, *** *p* < 0.001, ** *p* < 0.01, * *p* < 0.05, ns—not significant.

qPCR was used to evaluate the expression of genes related to obesity and anti-obesity to verify the anti-obesity activity of 11,14,17-ITA. Certain adipogenic markers’ amplified cDNA band densities were standardized in relation to GAPDH’s matching band density. At first, we checked different anti-obesity-related markers. Adiponectin expression was detected to be downregulated in OC by 0.63-fold and 0.61-fold compared to C and PC. This expression was upregulated gradually in three treated groups by 0.99-fold, 1.11-fold, and 1.47-fold. Similarly, PPAR-α, Lipoprotein Lipase (LPL), and Carnitine Palmitoyltransferase 1 (CPT1) were all highly expressed in the C and PC groups, but when we compared these expressions with the OC group, these were significantly downregulated. But the TX3 group showed a significantly upregulated expression (by 1.23-fold, 1.90-fold, and 1.42-fold) compared to the OC group, and when we compared the three treated groups, TX3 showed a more effective result than the other groups (Figure 6b–g). I have also checked two anti-inflammatory cytokines related to obesity, IL1R-a and IL-10, whose expression was gradually upregulated in TX1, TX2, and TX3 compared to OC.

We then determined the obesity gene expression level. In 11,14,17-ITA-containing TX3 groups, the expression of leptin and Sterol Regulatory Element Binding Protein 1c (SREBP-1c) was suppressed by 1.66-fold and 1.22-fold, respectively, and then OC. The mRNA level of the gene involved in lipogenesis (FAS) was upregulated in the OC group (by 1.26-fold, 1.17-fold) compared to C and PC. A high dose of 11,14,17-ITA markedly decreased this level by 1.17-fold. We investigated how this omega-3 fatty acid and HFD diet affected the expression of proinflammatory cytokine genes in adipose tissue. In adipose tissue, the HFD diet raised TNF-α expression, while fatty acid decreased TNF-α, IL1-β, and IL-6 expression (Figure 6h–m). TNF-α, IL1-β, and IL-6 expressions were lower in mice given 4 mg/kg B.W. fatty acid than in mice fed with an HFD diet.

### 3.5. Alteration of Proteins Involved with Obesity and Inflammation by 11,14,17-ITA

**Figure 7:**
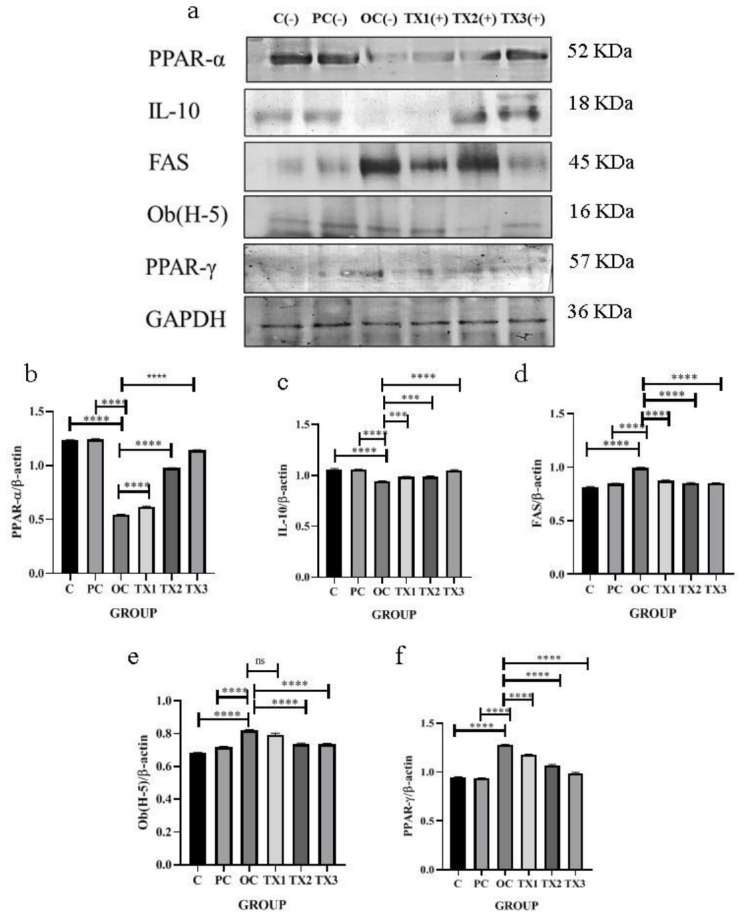
Isolated compound induced expression of obesity-related marker by Western blot analysis (**a**–**f**). Relative protein expression level of PPAR-α, IL-10, FAS, Ob(H-5), PPAR-γ, in adipose tissue of male mice in different experimental groups (n = 6) were adjusted to control protein expression level of β-actin. Values are expressed as mean ± SEM. One-Way ANOVA followed by Tukey’s multiple comparison test. **** *p* < 0.0001, *** *p* < 0.001, ns—not significant.

This research examined the potential influence of this fatty acid on obesity and inflammation related to obesity. The expression of PPAR-α and IL-10 was significantly decreased in the OC group compared to C and PC, but 11,14,17-ITA treatment markedly increased in vWAT (Figure 7b,c). This fatty acid administration gradually lowers the expression of FAS, Ob(H-5), and PPAR-γ in the TX1, TX2, and TX3 groups compared to OC (Figure 7d–f). In addition, 11,14,17-ITA plays an important role by inhibiting the synthesis of triglyceride in tissue as well as adipocyte cell differentiation.

## 4. Discussion

Obesity is linked to white adipose tissue macrophage infiltration and persistent subclinical inflammation [17,18,19,20]. This inflammatory response is responsible for the occurrence of different metabolic complications associated with obesity. Increased adipose gene expression is associated with elevated lipid profiles and long-term inflammatory markers such as TNF-α, IL-1β, IL-6, etc. Steatohepatitis, or ectopic fat buildup in the liver, results in liver failure. In this present study, we demonstrated that omega-3 fatty acid-based food can suppress HFD-induced obesity and adipocyte hypertrophy in Swiss albino male mice. Overfeeding is thought to be the primary cause of obesity in many cases, and treating obesity with a hypocaloric diet is a popular nutritional approach. A hypocaloric diet encourages weight loss and fat mobilization by creating a negative energy balance [21]. In this study, it was established that omega-3 fatty acids can trigger weight loss via stimulation and activation of adipocytokines rather than hunger suppression, as those treated mice consumed less food than C and PC mice. Addition of 20:3(n-3) 11,14,17-ITA at 2 and 4 mg/kg b.w. to HFD-fed mice dramatically reduced body weight growth as compared to OC without changing food intake, and there is no impact of the 1 mg/kg b.w. dose (Figure 1). There were notable reductions in visceral fat mass among the TX2 and TX3 in conjunction with the inhibition of body weight growth. Also, 20:3(n-3) 11, 14, 17-ITA fatty acid derived from *Setipinna phasa* oil has an effect on body weight gain comparable to the previous study [22]. Side by side, there is a similar effect on BMI after treatment with this fatty acid compared to OC (Figure 2); this is also comparable to my other study [12].

One typical metabolic consequence linked to obesity is hepatic steatosis. Unbalanced lipogenesis and lipid oxidation unique to the liver are the main causes of hepatic steatosis. In the context of obesity, there is growing evidence that the metabolic communication between the liver and adipose tissues may be a major factor in hepatic fat deposition. Few studies proved that obesity-related hepatic fat deposition was significantly worsened by adipose inflammation brought on by pro-inflammatory chemokines and cytokines, and HFD-induced hepatic steatosis could be completely prevented by reducing this inflammation [23,24]. The result of this study suggested that 20:3(n-3) 11,14,17-ITA plays a crucial role in combating lipid synthesis in the liver (Figure 3) and avoiding ectopic liver; this might be due to the suppression of inflammatory cytokines [25].

Adipocyte hypertrophy and hyperplasia are components of obesity, which is characterized by an abnormal buildup of lipids [26]. We performed histological section of vWAT and sWAT and then staining with two different stains. Notably, 20:3(n-3) 11, 14, 17—ITA treatment significantly reduced the size of fat droplets and total weight of WAT (Figure 4 and Figure 5). It might happen that anti-inflammatory cytokines are activated inside the fat cells due to the presence of this omega-3 fatty acid [17].

Previously, a few studies have already established that omega-3 fatty acids from dietary fish oil sources have a hypolipidemic effect [27,28]. Consequently, it is plausible that these bioactive metabolites present in marine sources affect lipid dynamics and further shield the treated mice from obesity. In this paper, we found that the effect of 20:3(n-3) 11,14,17-ITA on lowering the total lipid profile and increasing HDL is consistent with other studies (Table 3). The compound may have two potential mechanisms for the hypolipidemic impact that has been observed: either it interferes with the manufacture of cholesterol or it reduces the intestinal tract’s absorption of dietary cholesterol [29].

We assessed fasting blood glucose and total protein, considering the strong correlation between obesity and glycolipid and protein metabolism. This result showed that HFD food can lead to disruption of glucose metabolism. Treatment with this fatty acid significantly decreased serum glucose concentration and regulated glucose metabolism [30]. Significantly higher levels of protein were present in the C, PC, and treated groups compared to the OC group. This is comparable to a previous study [12].

The fatty acid supplementation did not adversely affect the liver or kidney at any dosage, which is crucial, suggesting that the dosages were both safe and effective for the intended course of treatment. This result was corroborated by another study [31].

The primary regulator of obesity is the gene adiponectin, which has a negative correlation with obesity. It affects circulation to regulate fat metabolism, fat oxidation, and glycolysis. Because of this, extra fat cannot be retained in adipose tissue; instead, it is burned to release energy from our bodies by the activation of this gene. The presence of long-chain omega-3 fatty acids in higher doses significantly upregulates the expression of this gene compared to the other group of treated mice (Figure 6b), and thereby minimizes the chances of gaining excess fat in the mice’s bodies. It is established by a few articles [12,32]. It is generally known that by blocking the lipogenic factor, PUFA and marine fish oil can cause the increased expression of PPAR-α [33]. In this study, 20:3(n-3) 11,14,17-ITA induces the expression of the PPAR-α marker (Figure 6c and Figure 7b) via encouraging the oxidation of fatty acids and inhibiting lipogenesis and activation of LPL. Some paper has reported that LPL gene expression was upregulated after treatment with EPA and DHA, which help hydrolyze the triglyceride that promotes the uptake of free fatty acids [34]. In this paper, it is established that this novel fatty acid can minimize the chances of weight gain in a person by upregulating this gene. In this study, CPT1 expression was also upregulated after treatment with this fatty acid at a higher dose. Through the translocation of fatty acids from the cytosol to the mitochondrial matrix, CPT1 inhibits the growth of white adipose tissue in the skin and in several organs. This study is comparable to another study [34]. EPA supplementation reduces the inflammatory response through suppression of the M1 macrophage marker [17,35]. In this study, it is established that a direct effect of 20:3(n-3) 11,14,17-ITA treatment induced expression of anti-inflammatory markers in the TX3 group; these are IL1R-a and IL-10. As we know, leptin resistance is common in obesity, and this expression is upregulated after treatment with marine fish oil and fish oil-derived fatty acids, as proven by a few articles [12,36,37]. Leptin and Ob(h-5) expression were downregulated after application of this omega-3 fatty acid at a higher dose. This might be due to the presence of this fatty acid through clearance of triglyceride from fat cells. SREBP-1c plays a critical role in the transcription of lipogenic genes, which controls the expression of fatty acid production indicators like FAS, which regulates fatty acid synthesis [35]. Our data indicated that fish oil-derived omega-3 fatty acid significantly decreased SREBP-1c and FAS mRNA in male Swiss mice and also suppressed the expression of protein activity of FAS. Chronic low-grade inflammation is positively correlated with obesity and abnormal fatty acid composition of the diet, all of which are detrimental to an individual’s health [38]. Adipose tissue deposition in the abdomen and in other organs caused by excess ingestion of a high-fat diet by an individual is stimulated to release more inflammatory cytokines in their body, like TNF-α, IL1β, and IL-6 [39]. In our study, it was discovered that obese mice had a notable rise in TNF-α mRNA expression. Treatment with a higher dose of PUFA in the supplement reduced this increase. Also, we saw that the mice in the obese group secreted more IL-1β and IL-6 and that the fatty acid obtained from marine fish oil tended to inhibit this rise. As we know, PUFA reduces inflammatory cytokine production, which is associated with obesity [35,37]. Many forms of omega-3 fatty acids bind to PPAR-γ, which is crucial for controlling adipogenesis through adipocyte differentiation [40]. In our study, it has been proven that this long-chain omega-3 fatty acid lowers the expression of this protein at a 4 mg/kg b.w. dose compared to the other two treated groups as well as the OC group.

## 5. Conclusions

Taken together, all the data from our study concluded that treatment of obese Swiss mice with marine *Setipinna phasa* oil-derived fatty acid, i.e., 20:3(n-3) 11,14,17-ITA, is effective in decreasing total body weight, BMI, MASLD, adiposity of WAT in subcutaneous and visceral parts, hypertrophy of adipocytes, serum lipid profile, liver toxicity, and other biochemical parameters. It also improved fatty acid β-oxidation by upregulating genes and also upregulating a few adipokines. It lowers the risk of obesity and obesity-associated metabolic disorder by suppressing these enzymes: SREBP1-c and FAS. Obesity-induced inflammation is also prevented by lowering the expression of TNF-α, IL1β, and IL-6. Further, 20:3(n-3) 11, 14, 17 ITA is able to maintain cell homeostasis by energy production via activation of those markers involved in fatty acid oxidation (PPAR-α and CPT1). They promote the breakdown of fatty acids for energy production. The broken fatty acids are then transported to the mitochondria, where they then undergo further breakdown (citric acid cycle).

## Figures and Tables

**Table 1:** Study design and animal treatments based on a high-fat diet (HFD) and no-fat diet (NFD) in a Swiss model.

Group (*n* = 6)	Feed Type	Treatment
Control (C)	NFD	0.2 mL 0.5% methylcellulose
Positive Vehicle Control (PC)	NFD	2 µL olive oil
Obese Control (OC)	HFD	0.2 mL 0.5% methylcellulose
Treatment 1 (TX1)	HFD	1 mg/kg of B.W. ITA with 2 µL olive oil
Treatment 2 (TX2)	HFD	2 mg/kg of B.W. ITA with 2 µL olive oil
Treatment 3 (TX3)	HFD	4 mg/kg of B.W. ITA with 2 µL olive oil

**Table 2:** Primers for quantitative PCR.

Gene	Forward (5′ to 3′)	Reverse (5′ to 3′)
Adiponectin	GTCAGTGGATCTGACGACACCAA	ATGCCTGCCATCCAACCTG
LPL	CCACAGCAGCAAGACCTTC	AGGGCGGCCACAAGTTTG
PPAR-α	CCTGAACATCGAGTGTCGAATAT	GTTCTTCTTCTGAATCTTGCAGCT
CPT1	GTGACTGGTGGGAGGAATAC	GAGCATCTCCATGGCGTAG
IL1R-a	GCAGCACAGGCTGGTGAATGAC	TGCCCCCGTGGATGCCCAAG
IL-10	TCTCCGAGATGCCTTCAGCAGA	TCAGACAAGGCTTGGCAACCCA
LEPTIN	CAAGCAGTGCCTATCCAGA	AAGCCCAGGAATGAAGTCCA
SREBP-1C	ACGGAGCCATGGATTGCACA	AAGGGTGCAGGTGTCACCTT
FAS	TGCTCCCAGCTGCAGGC	GCCCGGTAGCTCTGGGTGTA
TNF-α	TTCTGTCTACTGAACTTCGGGGTGATCGGTTCC	GTATGAGATAGCAAATCGGCTGACGGTGTGGG
IL-1β	ATGGCAACTGTTCCTGAACTCAACT	CAGGACAGGTATAGATTCTTTCCTTT
IL-6	TCAACTTCTCCAGCGTGATG	TCTTTCCCTCTTTTCCTCC
GAPDH	GGTGAAGGTCGGAGTCAACG	GTGAAGACGCCAGTGGACTC

**Table 3:** The effect of isolated compound on blood biochemistry in different experimental groups. Values are expressed as mean ± SEM (n = 6). One-Way ANOVA followed by Tukey’s multiple comparison test. **** *p* < 0.0001, ** *p* < 0.01, ns—not significant.

Biochemical Parameters	C	PC	OC	TX1	TX2	TX3
TC mg/dL	99.15 ± 0.64	99.27 ± 0.51	162.9 ± 0.64 ****	121.6 ± 0.49 ****	114.4 ± 0.54 ****	99.32 ± 0.69 ****
TG mg/dL	76.58 ± 0.43	76.50 ± 0.54	194.6 ± 1.62 ****	156.1 ± 1.78 ****	95.63 ± 1.91 ****	72.60 ± 1.21 ****
VLDL mg/dL	15.32 ± 0.09	15.33 ± 0.11	38.90 ± 0.32 ****	31.22 ± 0.35 ****	19.12 ± 0.38 ****	14.50 ± 0.24 ****
LDL mg/dL	17.35 ± 2.28	11.33 ± 1.57	84.72 ± 0.73 ****	46.45 ± 0.93 ****	34.53 ± 1.19 ****	18.25 ± 1.35 ****
HDL mg/dL	66.48 ± 1.95	72.62 ± 1.52	39.27 ± 0.83 ****	43.88 ± 0.46 **^ns^**	60.75 ± 0.51 ****	66.55 ± 0.95 ****
Serum glucose mg/dL	102.9 ± 0.64	104.8 ± 0.57	152.2 ± 2.15 ****	145.2 ± 1.20 **	97.68 ± 0.80 ****	105.3 ± 93 ****
Serum proteins g/dL	7.33 ± 0.04	8.25 ± 0.19	3.12 ± 0.35 ****	5.15 ± 0.20 ****	6.22 ± 0.03 ****	8.23 ± 0.05 ****
Uric acid mg/dL	3.69 ± 0.23	3.41 ± 0.06	9.84 ± 0.17 ****	7.03 ± 0.20 ****	4.80 ± 0.03 ****	3.19 ± 0.05 ****
SGOT U/L	12.58 ± 0.44	14.38 ± 0.31	46.38 ± 0.18 ****	32.52 ± 0.57 ****	26.67 ± 0.47 ****	19.90 ± 0.46 ****
SGPT U/L	14.23 ± 0.26	17.68 ± 0.46	47.33 ± 0.56 ****	34.63 ± 0.92 ****	27.37 ± 0.13 ****	16.66 ± 0.51 ****
ALP U/L	92.11 ± 0.90	82.29 ± 0.56	261.4 ± 2.55 ****	234.6 ± 0.57 ****	180.8 ± 1.89 ****	146.4 ± 2.05 ****

## Data Availability

Dataset available on request from the authors.

## Abbreviations

The following abbreviations are used in this manuscript:

11,14,17-ITA	Icosa-11,14,17-trienoic acid/Methyl cis-11,14,17-Icosatrienoate
ALP	Alkaline Phosphatase
B.W.	Body Weight
BMI	Body Mass Index
cDNA	Complementary Deoxyribonucleic Acid
CPT1	Carnitine Palmitoyltransferase 1
CVD	Cardiovascular diseases
DHA	Docosahexaenoic Acid
EPA	Eicosapentaenoic Acid
FAS	Fatty Acid Synthase
FFA	Free Fatty Acids
GAPDH	Glyceraldehyde-3-Phosphate Dehydrogenase
GODPOD	Glucose Oxidase-Peroxidase
HDL	High Density Lipoprotein
HE	Hematoxylin and Eosin
HFD	High-Fat Diet
IL-1, IL-6, IL1-β, IL-10	Interleukin
IL1R-a	Interleukin-1 Receptor Antagonist
LDL	Low Density lipoprotein
LPL	Lipoprotein Lipase
MASLD	Metabolic Dysfunction-Associated Steatotic Liver Disease
MCP-1	Monocyte Chemotactic Protein 1
MUFA	Monounsaturated Fatty Acid
NAFLD	Non-Alcoholic Fatty Liver Disease
NFD	No-Fat Diet
PCR	Polymerase Chain Reaction
PPAR-α	Peroxisome Proliferator-Activated Receptor α
PPARγ	Peroxisome Proliferator-Activated Receptor γ
PUFA	Polyunsaturated Fatty Acid
RNA	Ribonucleic Acid
SDS-PAGE	Sodium Dodecyl Sulphate-Polyacrylamide Gel Electrophoresis
SFA	Saturated Fatty Acid
SGOT	Serum Glutamic-Oxaloacetic Transaminase
SGPT	Serum Glutamic Pyruvic Transaminase
SREBP-1c	Sterol Regulatory Element Binding Protein 1c
sWAT	Subcutaneous White Adipose Tissue
T2DM	Type II Diabetes Mellitus
TC	Total cholesterol
TG	Triglyceride
TNF-α	Tumor Necrosis Factor- α
VLDL	Very Low-Density Lipoprotein
vWAT	Visceral White Adipose Tissue
WAT	White Adipose Tissue
